# Urine Cadmium as a Risk Factor for Osteoporosis and Osteopenia: A Meta-Analysis

**DOI:** 10.3389/fmed.2021.648902

**Published:** 2021-04-16

**Authors:** Dong Li, HaoJie Lin, Min Zhang, Jing Meng, LiYou Hu, Bo Yu

**Affiliations:** ^1^The Chinese Medicine College, Shandong University of Traditional Chinese Medicine, Jinan, China; ^2^Jinan Blood Supply and Security Center, Jinan, China; ^3^Department of Nursing, Affiliated Hospital of Shandong University of Traditional Chinese Medicine, Jinan, China; ^4^Department of Orthopedics, Affiliated Hospital of Shandong University of Traditional Chinese Medicine, Jinan, China

**Keywords:** cadmium, osteoporosis, osteopenia, meta-analysis, risk factor

## Abstract

**Background:** As society ages, the incidence of osteoporosis increases. In several studies, cadmium (Cd) is thought to be related to osteoporosis. However, there are conflicting reports about the relationship between Cd and the risk of osteoporosis and osteopenia. Therefore, the purpose of this meta-analysis was to explore the relationship between Cd and osteoporosis and osteopenia.

**Methods:** Through a review of the literature, articles published in PubMed as of December 2020 were identified and the references of related publications and reviews were reviewed. Ultimately, 17 eligible articles were selected to determine the relationship between blood and urine Cd concentrations for the risk of osteoporosis or osteopenia. In this study, we performed a classification analysis, heterogeneity test, subgroup analysis, and evaluated publication bias.

**Results:** A total of 17 studies were included, including seven on blood Cd and 10 on urine Cd. By combining the odds ratio (OR) and 95% confidence interval (CI) for the lowest and highest categories, the odds ratio of blood Cd concentration that increased the risk of osteoporosis or osteopenia was OR 1.21 (95% CI: 0.84–1.58) and that of urine Cd concentration that increased the risk of osteoporosis or osteopenia was OR 1.80 (95% CI: 1.42–2.18), and the results of the subgroup analysis were also consistent.

**Conclusions:** Our research indicates that while urine cadmium (Cd) concentration may be related to increased risk of osteoporosis and osteopenia, blood Cd concentration may not. Therefore, compared to blood Cd concentration, urine Cd concentration may be more reliable as a risk factor for osteoporosis and osteopenia. This result should be interpreted with caution. Currently. research on the relationship between Cd concentration and osteoporosis and osteopenia is limited, thus, further large, high-quality prospective studies are required to elucidate the relationship between Cd concentration and osteoporosis and osteopenia.

## Introduction

Osteoporosis is a systemic bone disease characterized by decreased bone mineral density, bone microstructure destruction, and increased risk of fragility fractures. Due to the high morbidity and mortality of diseases such as osteoporosis, fragility fractures, and other diseases, it has become a public health problem that needs to be solved urgently (1, 2). Some metals such as zinc, iron, and copper are closely related to human bones and are necessary to maintain normal physiological functions. However, heavy metals have been reported as risk factors for degenerative diseases such as osteoporosis and associated fractures (3, 4).

Cadmium (Cd) is a toxic non-essential transition metal. With the acceleration of global industrialization, Cd and its inorganic compounds are widely used in the manufacturing process of electroplating, batteries, pigments, plastics, and alloys. A large amount of Cd will enter the soil and, ultimately, the human body through contaminated food and water (5). Cd accumulates in plants and animals, and its half-life is ~10–30 years. Epidemiological data indicate that occupational and environmental Cd exposure may be related to various types of cancer, and Cd may be a risk factor for osteoporosis (6). A number of animal studies have shown that Cd can directly affect bone density by stimulating osteoclast differentiation and activity (7) and can indirectly affect bone health by affecting other organ systems, such as the gastrointestinal tract, thyroid, and especially, the kidneys (8, 9). However, the results of investigations on the relationship among human Cd intake, body Cd concentration, and osteoporosis are not consistent. Songprasert et al. reported that excessive exposure and intake of Cd will cause bone density reduction and osteoporosis (10). Li X et al. also reached the same conclusion (11). However, Trzcinka-Ochocka suggested that Cd has no correlation with osteoporosis and bone density (12). Therefore, clarifying the relationship between Cd concentration and osteoporosis or osteopenia is helpful in the formulation of clinical policies and guidelines. However, there is currently no relevant meta-analysis to explain the relationship between blood and urine Cd concentrations and the risk of osteoporosis and osteopenia.

Therefore, the purpose of this meta-analysis was to explore the relationship between blood and urine Cd concentrations and the risk of osteoporosis or osteopenia.

## Methods

Ethical approval and written informed consent from patients were not necessary because our study was based on summaries and analyses of results of existing studies.

### Search Strategy and Data Sources

Free keywords were used to search for articles published in PubMed till December 2020. The search words used were “cadmium,” “osteoporosis,” “osteopenia,” and “bone density.” In addition, in order to obtain further relevant literature, we manually searched the references for related articles.

### Selection Criteria

The articles were independently selected and commented on by two authors. First, the title and abstract were filtered based on the relevance of the topic. After reading the abstract, the full text was screened and articles that will eventually be included in the meta-analysis were selected. Articles that met the inclusion criteria were independently selected by two authors. When it was unclear whether an article should be included, a discussion was conducted with the third author to reach a consensus.

The inclusion criteria were as follows: (1) studies including human subjects; (2) observational studies; (3) studies that reported the relationship between blood or urine Cd concentration and osteoporosis or bone mass loss, and (4) studies that calculated and reported relative risk (RR), odds ratio (OR), or hazard ratio (HR) and 95% confidence interval (CI) values.

The exclusion criteria were as follows: (1) Animal experiments; (2) *in vitro* or laboratory studies; and (3) comments or case reports.

### Data Extraction and Quality Assessment

Two examiners used standardized data collection forms to extract data independently. These differences were resolved through discussions with other investigators and referenced to the original article. The data extracted from each study included the first author's last name, publication year, study type, average age, male to female ratio, sample size, study country, bone density measurement method, blood or urine Cd concentration, adjusted variables, and the corresponding 95% CIs-OR estimate. If the OR value of different potential confounding factors was high, the OR value extracted reflected the maximum control of the potential confounding factors. When required, the authors of the preliminary study were contacted for more information.

### Statistical Analyses

Research data consisting of the OR of blood or urine Cd concentration and the risk of osteoporosis or osteopenia were included for analysis, and the size of the impact was expressed as 95% CI; a random-effects model was implemented (13). Cochran Q statistics and *I*^2^ statistics were used to assess the heterogeneity between studies (14). *I*^2^ values of 25%, 50, and 75% were considered low, medium, and high heterogeneity, respectively (15). Subgroup analysis separately assessed the relationship between blood and urine Cd concentrations and related research characteristics (sex and degree of osteoporosis) of the risk of osteoporosis or osteopenia, as a possible source of heterogeneity. Funnel chart asymmetry was used to test publication bias, and Begg's and Egger's tests were employed to measure funnel chart asymmetry (16). A “cut and fill” assessment was conducted to further evaluate the possible impact of publication bias in our meta-analysis. This method reflects the empirical research that causes funnel graph asymmetry by conservatively attributing to hypothetical negative unpublished research (17). Osteoporosis was classified as normal (T-score > −1.0), osteopenia (−2.5 ≤ T-score ≤ −1.0), and osteoporosis (T-score < −2.5). All statistical analyses were performed using STATA 12 (StataCorp, College Station, TX, USA).

## Results

### Search Results

**Figure 1:**
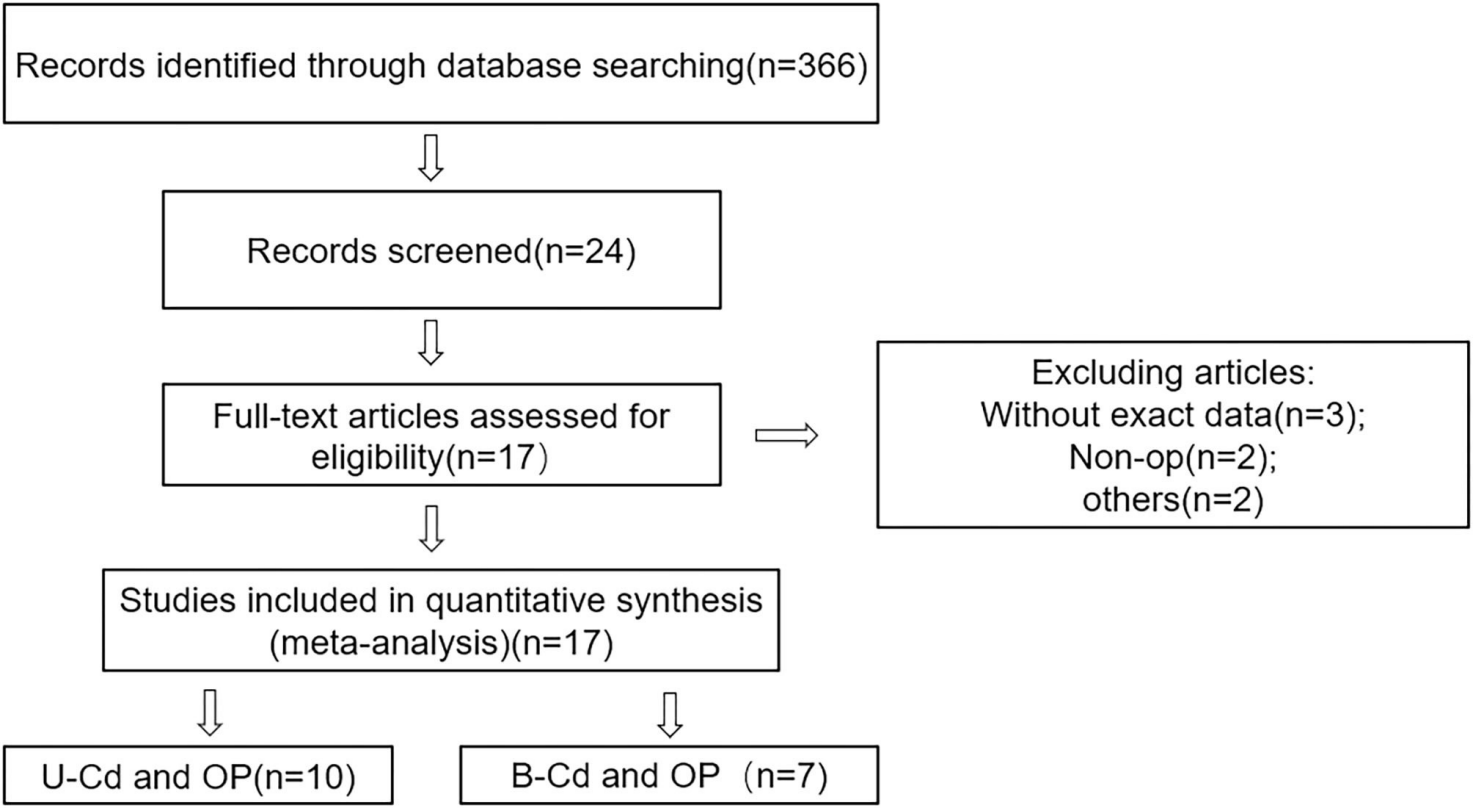
Flowchart of the studies selection.

Figure 1 shows the process of document screening, research selection, and exclusion. The initial database search included 336 articles. After reading the abstract and title, 342 articles were excluded. The quality of the remaining 24 articles was evaluated, and seven articles that did not meet the inclusion criteria were excluded. Finally, 17 articles were selected for the meta-analysis, of which seven were focused on blood Cd concentrations (18–24) and 10 on urine Cd concentrations (25–34).

### Research Characteristics

A total of 29,488 people from 17 studies were included in the analysis. Seven studies measured blood Cd concentration, involving a total of 6,864 subjects, with 4,191 men and 2,673 women. Only two articles were related to osteoporosis and the remaining five articles were related to osteoporosis and osteopenia. Meanwhile, 10 studies measured urine Cd concentration, involving 22,624 people, with 2,229 males and 9,417 females. Six articles involved osteoporosis, two involved osteopenia, and two involved osteoporosis and osteopenia. The risk estimates provided by most studies were adjusted for age, sex, smoking, body mass index, physical activity, and weight.

Tables 1, 2 summarize the characteristics of the study and participants.

**Table 1:** Summary characteristics of studies and participants.

**References**	**Measured**	**Study type**	**Age (years)**	**Male-female ratio**	**Number**	**Country**	**BMD measured**
Lim et al. (24)	B-Cd	Cross-sectional	>18	1,229/1,200	2,429	Korea	
Burm et al. (23)	B-Cd	Cross-sectional	40.3	1,275/–	1,275	Korea	Dual-energy X-ray
Choi and Han (22)	B-Cd	Cross-sectional	58.81	1,089/–	1,089	Korea	Dual-energy X-ray
Chen et al. (21)	B-Cd	Cross-sectional	Women Control area 51.9	119/202	321	China	Dual energy X-ray
			Women Polluted area 58.7				
			Men Control area 57.2				
			Men Polluted area 64.2				
Pollack et al. (20)	B-Cd	Cross-sectional	27.4	–/248	248	America	Dual energy X-ray
Cho et al. (19)	B-Cd	Cross-sectional	62.1 ± 8.2	–/481	481	Korea	Dual energy X-ray
Alfvén et al. (18)	B-Cd	Cross-sectional	Men 54	479/542	1,021	Sweden	Dual energy X-ray
			Women 52				
Lv et al. (32)	U-Cd	Cross-sectional	Non-Cd-polluted area 56.9	511/605	1,116	China	Dual energy X-ray
			Cd-polluted area 55.8				
Van Larebekea et al. (34)	U-Cd	Cross-sectional	50–65	–/808	808	Belgium	Dual energy X-ray
Kim et al. (31)	U-Cd	Cross-sectional	Male 63.8	456/630	1,086	Korea	Ultrasound bone densitometer
			Female 65.2				
Engström et al. (30)	U-Cd	Cross-sectional	<70	–/2,688	2,688	Sweden	Dual-energy X-ray
Shin et al. (29)	U-Cd	Prospective cohort		357/447	804	Korea	Dual-energy X-ray
Wu et al. (33)	U-Cd	Cross-sectional	30–90		10,978	America	
Nawrot et al. (28)	U-Cd	Cross-sectional	45	83/–	83	Belgium	Dual-energy X-ray
Gallagher et al. (27)	U-Cd	Cross-sectional	67	–/3,207	3,207	America	Dual-energy X-ray
Wang et al. (26)	U-Cd	Cross-sectional	Male control 54.3	302/488	790	China	SPA-4 single-photon absorptiometry
			Male moderate 51.1				
			Male heavy 55.4				
			Female control 50.0				
			Female moderate 51.3				
			Female heavy 52.4				
Alfvén et al. (25)	U-Cd	Cross-sectional	Environmentally exposed	520/544	1,064	Sweden	Dual-energy X-ray
			Male 52.0				
			Female 51.4				
			Occupationally exposed				
			Male 58.4				
			Female 56.5				

**Table 2:** Summary characteristics of studies.

**B-Cd**							
**References**	**Measured**	**Type**		**B-Cd(μg/g)**	**or**	**95% CI**	**Adjustment**
Lim et al. (24)	Graphite furnace atomic absorption spectrometry		Q1	0.66	1		Age, sex, lifestyle behaviors (smoking status, alcohol drinking, and living region). sociodemographic factors (educational level, occupation and family income).
			Q2	0.825	0.99	(0.77–1.26)	
			Q3	1.2145	1.01	(0.79–1.31)	
			Q4	1.439	1.8	(1.35–2.4)	
Burm et al. (23)	Atomic absorption spectrophotometry	Total femur		0.83	1.81	(1.07–3.07)	Age, body mass index, height, household income, alcohol consumption, hypertention, diabetes mellitus, exercise and urinary cotinine.
		Lumbar spine		0.83	1.17	(0.87–1.57)	
		Femoral neck		0.83	1.49	(1.1–2.03)	
Choi and Han (22)	Graphite furnace atomic absorption spectrometry	Non-Obese	Q1	1	1		Age, BMI (as a continuous variable), serum creatinine (as a continuous variable), vitamin D deficiency [serum 25(OH)D <20 ng/mL], smoking (current smoker vs. non-smoker), alcohol drinking (>7 drinks of alcoholic beverage per time, twice or more in a week: yes or no) and physical activity (vigorous physical activity for more than 20 min per time, three times or more in a week: yes or no).
		Non-Obese	Q2	1.25	0.83	(0.51–1.36)	
		Non-Obese	Q3	1.5	0.72	(0.42–1.23)	
		Obese	Q1	1	1		
		Obese	Q2	1.25	2.36	(0.92–6.08)	
		Obese	Q3	1.5	5.71	(1.99–16.38)	
Chen et al. (21)	Graphite furnace atomic absorption spectrometry	Male		2	0.93	(0.3–2.74)	Age, weight, height, smoking, alcohol and menopause status (women)
		Female		2	2.5	(1.11–5.43)	
Pollack et al. (20)	Inductively coupled plasma mass spectrometry	Whole body		0.36	0.76	(0.36–1.61)	Age (continuous), race (white, black, Asian, other), parity, average caloric intake (continuous), age at menarche (continuous)
		Total hip		0.36	0.98	(0.89–1.07)	
		Lumbar spine		0.36	1.17	(0.56–2.46)	
		Wrist		0.36	0.91	(0.43–1.94)	
Cho et al. (19)	Atomic absorption spectrophotometry		Q1	1	1		Intake of caloric energy and calcium, fish consumption, and vitamin D level in addition to the corrections included in model 1. Pb, lead; Hg, mercury; Cd, cadmium; As, arsenic.
			Q2	1.19	1.22	(0.65–2.29)	
			Q3	1.58	1.27	(0.68–2.39)	
			Q4	1.78	0.96	(0.51–1.81)	
Alfvén et al. (18)	Inductively coupled plasma mass spectrometry		Q1	0.56	1		Weight, smoking,
			Q2	0.84	2	(1.1–3.9)	
			Q3	1.12	2.9	(1.4–5.8)	
**U-Cd**							
**References**	**Measured**	**Type**		**U-Cd (μg/g)**	**OR**	**95% CI**	**Adjustment**
Lv et al. (32)	Inductively coupled plasma mass spectrometry	Total	Q1	2.05	1	1	Age, gender, BMI, serum albumin, urinary Ca, and urinary U-Alb.
		Total	Q2	3.01	3.07	(1.77–5.33)	
		Total	Q3	6.43	4.63	(2.68–7.98)	
		Total	Q4	8.89	9.15	(5.26–15.94)	
		Nonsmokers	Q1	2.05	1		
		Nonsmokers	Q2	3.01	1.85	(0.89–3.86)	
		Nonsmokers	Q3	6.43	3.27	(1.6–6.68)	
		Nonsmokers	Q4	8.89	9.29	(4.56–18.93)	
Van Larebekea et al. (34)	Inductively coupled plasma mass spectrometry	Female		0.625	1.26	(0.97–1.63)	BMI, education status, and exercise level
Kim et al. (31)	Atomic absorption spectrophotometer	Male	Q1	≤5	1		Age, smoking status, alcohol intake, BMI, diabetes, hypertension, and menopause (only females).
		Male	Q2	>5	3.12	(1.36–7.14)	
		Female	Q1	≤5	1		
		Female	Q2	>5	2.8	(1.6–4.9)	
		Total	Q1	≤5	1		Age, sex (only total subjects), smoking status, alcohol, intake, BMI, diabetes, hypertension, and menopause (only females).
		Total	Q2	>5	1.54	(1.05–2.25)	
Engström et al. (30)	Inductively coupled plasma mass spectrometry	Femoral neck	Q1	0.5	1		Age (years), education (≤9 and >9 years; yes/no), height (cm), total fat mass (kg), lean body mass (kg), parity (0–6), use of postmenopausal hormones (yes/no), ever use of corticosteroids (yes/no), total physical activity (MET-hours/day), smoking status (never/ever), alcohol intake (g ethanol/day), inflammatory joint diseases (yes/no), kidney diseases (yes/no), liver diseases (yes/no), malabsorption (yes/no).
		Femoral neck	Q2	0.625	2.17	(1.51–3.11)	
		Femoral neck	Q3	0.75	2.45	(1.51–3.97)	
		Total hip	Q1	0.5	1		
		Total hip	Q2	0.625	1.49	(0.75–2.97)	
		Total hip	Q	0.75	3.01	(1.41–6.43)	
		Lumbar spine	Q1	0.5	1		
		Lumbar spine	Q2	0.625	1.3	(0.91–1.86)	
		Lumbar spine	Q3	0.75	1.97	(1.24–3.14)	
		Hip or spine	Q1	0.5	1		
		Hip or spine	Q2	0.625	1.61	(1.2–2.16)	
		Hip or spine	Q3	0.75	1.95	(1.3–2.93)	
Shin et al. (29)	Atomic absorption spectroscopy	Male		0.5	1		
		Male		0.75	1.18	(0.57–2.44)	
		Male		1	2.92	(1.51–5.64)	
		Female		0.5	1		
		Female		0.75	1.29	(0.49–3.36)	
		Female		1	3.37	(1.09–10.38)	
Nawrot et al. (28)	Inductively coupled plasma mass spectrometry		Q1	0.51	1		Age, age squared, and current smoking
			Q2	1.195	4.8	(0.88–29.1)	
			Q3	1.88	9.9	(1.8–55.2)	
Wu et al. (33)	Atomic absorption spectrometry	Opo-total	Q1	1	1		Age (continuous), sex (men vs. women, not for sex subgroup analysis), ethnicity or race (non-Hispanic black and Mexican American compared with non-Hispanic white, not for race subgroup analysis), BMI (continuous), calcium intake (continuous), and physical activity
		Opo-total	Q2	1.5	1.78	(1.26-2.52)	
		Opo-total	Q3	2	3.8	(2.36-6.14)	
		Opo-male	Q1	1	1		
		Opo-male	Q2	1.5	2.11	(1.05–4.22)	
		Opo-male	Q3	2	5.36	(2.31–12.64)	
		Opo-female	Q1	1	1		
		Opo-female	Q2	1.5	1.6	(1.12–2.29)	
		Opo-female	Q3	2	3.36	(1.86–6.04)	
		Ope-total	Q1	1	1		
		Ope-total	Q2	1.5	1.49	(1.24–1.8)	
		Ope-total	Q3	2	2.05	(1.52–2.78)	
		Ope-male	Q1	1	1		
		Ope-male	Q2	1.5	1.46	(1.03–2.07)	
		Ope-male	Q3	2	2.52	(1.24–5.11)	
		Ope-female	Q1	1	1		
		Ope-female	Q2	1.5	1.41	(1.13–1.75)	
		Ope-female	Q3	2	1.81	(1.21–2.71)	
Gallagher et al. (27)	Atomic absorption spectrometry	Hip BMD	Q1	0.5	1		Age, race, income, ever-smoker, underweight, and survey-respondent–reported physician diag- nosis of renal impairment.
		Hip BMD	Q2	0.75	1.43	(1.02–2)	
		Hip BMD	Q3	1	1.4	(0.97–2.03	
		Physician diagnosed	Q1	0.5	1		
		Physician diagnosed	Q2	0.75	1.46	(0.84–2.55)	
		Physician diagnosed	Q3	1	1.47	(0.81–2.66)	
Wang et al. (26)	Atomic absorption spectrophotometry	Male	Q1	1.58	1		
		Male	Q2	2.27	0.75	(0.1–4.5)	
		Male	Q3	9.2	1.72	(0.5–5.9)	
		Females	Q1	1.79	1		
		Females	Q2	4.45	1.38	(0.7–2.8)	
		Females	Q3	12.86	2.09	(1.1–4)	
Alfvén et al. (25)	Inductively coupled plasma mass spectrometry		Q1	0.5	1		
			Q2	1.75	1.2	(0.82–1.8	
			Q3	3	2.5	(1.2–5.2)	

### Blood Cadmium Concentration Level and the Risk of Osteoporosis or Osteopenia

**Figure 2:**
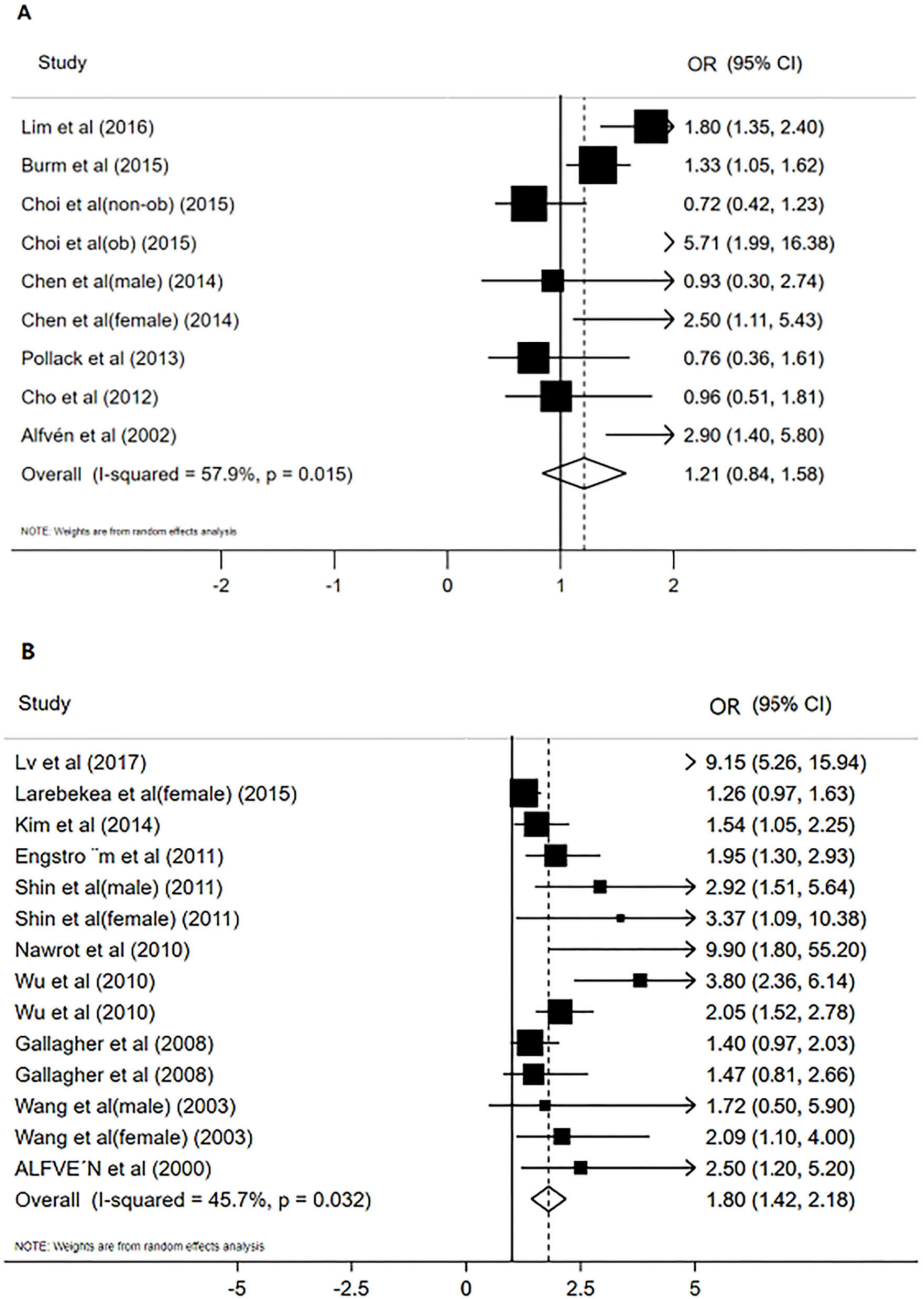
The forest plot for studies on the concentration of blood Cd and osteoporosis or osteopenia **(A)**, Urinary Cd concentration and osteoporosis or osteopenia **(B)**.

**Figure 3:**
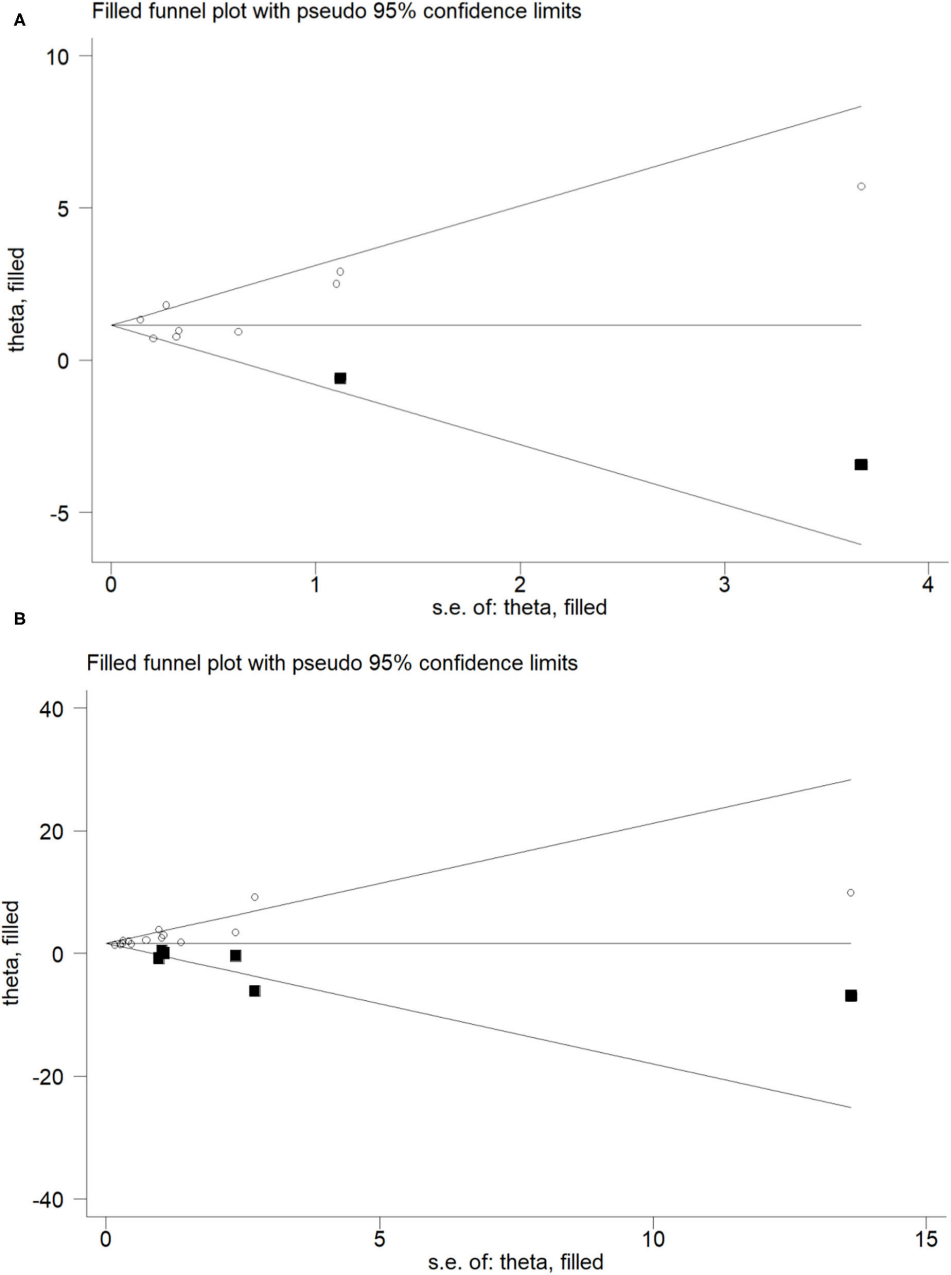
Trim and fill funnel plot for meta-analysis of the association between Blood Cd concentration and osteoporosis or osteopenia **(A)**, Urinary Cd concentration and osteoporosis or osteopenia **(B)**.

Figure 2A shows the results of blood Cd meta-analysis. Blood Cd concentration increased the risk of osteoporosis or osteopenia (OR = 1.21, 95% CI: 0.84–1.58), and the heterogeneity between different studies was moderate (*I*^2^ = 57.9%, *P* = 0.015). The comprehensive estimate of the risk of blood Cd concentration events did not change substantially without any research conducted through sensitivity analysis (Figure 3A).

### Urinary Cadmium Concentration Level and the Risk of Osteoporosis or Osteopenia

Figure 2B shows the results of the urine Cd meta-analysis. Urinary Cd concentration increased the risk of osteoporosis or osteopenia (OR = 1.80, 95% CI: 1.42–2.18). The heterogeneity between different studies was moderate (*I*^2^ = 45.7%, *P* = 0.032). The comprehensive estimate of the risk of urinary Cd concentration events did not change substantially after excluding any research conducted through sensitivity analysis (Figure 3B).

### Publication Bias

**Figure 4:**
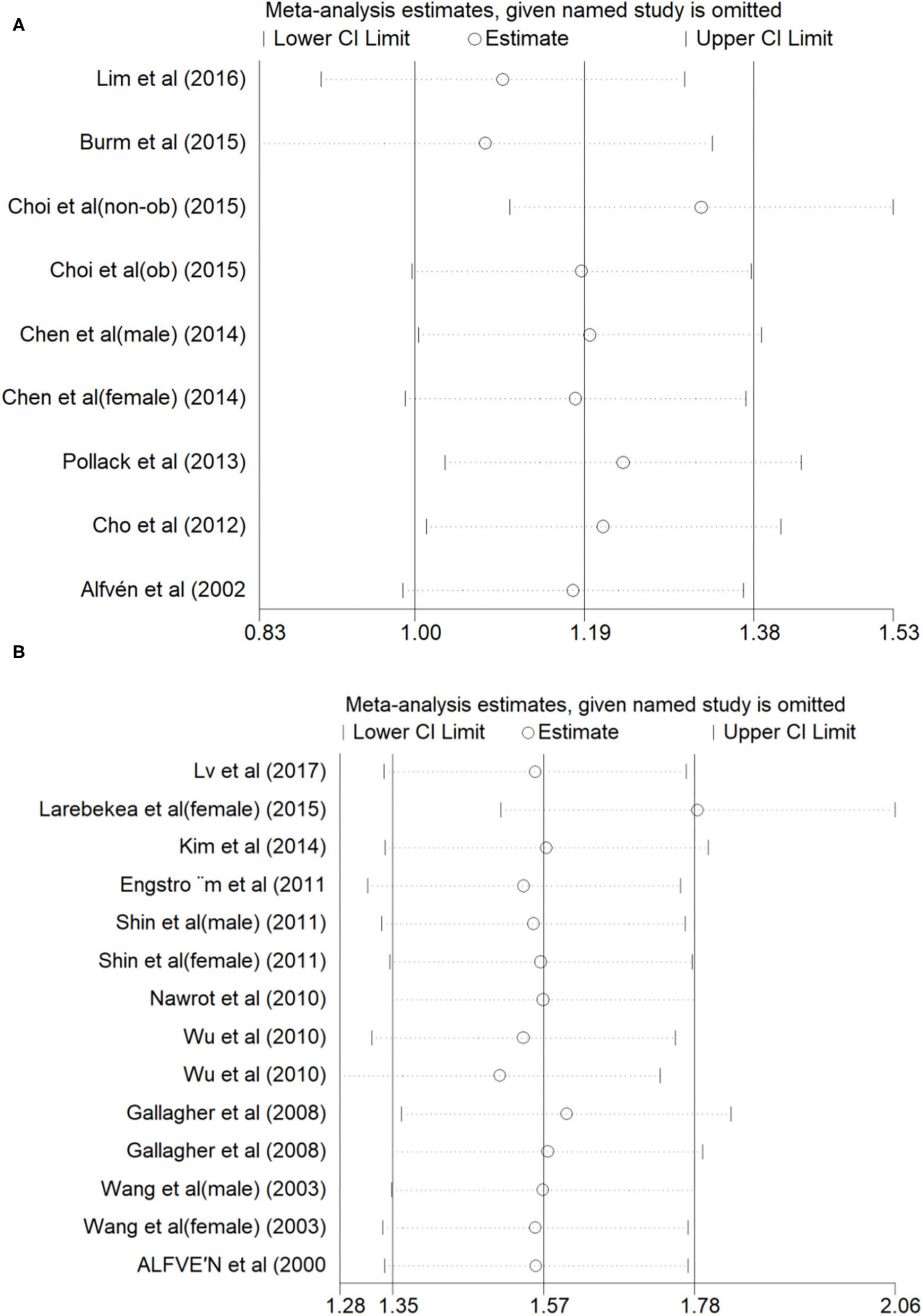
Sensitivity analysis of Blood Cd concentration and osteoporosis or osteopenia **(A)**, Urinary Cd concentration and osteoporosis or osteopenia **(B)**.

For the relationship between blood Cd concentration and osteoporosis and osteopenia, the Begg's test (*P* = 0.297, *z* = 1.04) and Egger's test (*P* = 0.396) showed no publication bias, whereas for the relationship between urine Cd concentration and osteoporosis and osteopenia, the Begg's test (*P* = 0.021, *z* = 2.31) and Egger's test (*P* = 0.000) showed publication bias. In order to further evaluate publication bias, we adopted the method of pruning and filling. Through computer filling, the results after correction for publication bias did not change (Estimate = 1.600; 95% CI: 1.166–2.034) (Figure 4).

### Subgroup Analysis

The correlation between blood Cd concentration and osteoporosis and osteopenia was analyzed by subgroup analysis. The results are shown in Table 3. Sex was evaluated as a source of heterogeneity. Blood Cd concentration was associated with an increased risk of osteoporosis and osteopenia for both males and females (male OR = 1.05, 95% CI: 0.54–1.57, *I*^2^ = 59.9%, *P* = 0.058; female OR = 0.94, 95% CI: 0.44–1.44, I^2^=13.8%, *P* = 0.313). In addition, a subgroup analysis of the degree of osteoporosis was conducted, with the following categories: normal (T-score > −1.0), osteopenia (−2.5 ≤ T-score ≤ −1.0), and osteoporosis (T-score < −2.5). Blood Cd concentration was associated with an increased risk of osteoporosis (T-score ≤ −1.0, OR = 1.38, 95% CI: 0.96–1.81, *I*^2^ = 48.5%, *P* = 0.084) and osteopenia (T-score < −2.5, OR = 0.81, 95% CI: 0.43–1.19, *I*^2^ = 7.9%, *P* = 0.338).

**Table 3:** Subgroup analysis to investigate differences between studies included in meta-analysis.

**Type**	**studies**	**OR (95% CI)**	***I*^**2**^**	***P*-value**
**B-Cd**				
Osteoporosis and Osteopenia	5	1.38 (0.96–1.81)	48.5%	0.084
Osteoporosis	2	0.81 (0.43–1.19)	7.9%	0.338
**B-Cd**				
Male	3	1.05 (0.54–1.57)	59.9%	0.058
Female	3	0.94 (0.44–1.44)	13.8%	0.313
**U-cd**				
Osteoporosis	8	1.86 (1.36–2.36)	47.7%	0.033
Osteopenia	2	2.03 (1.38–2.69)	0.0%	0.657
**U-Cd**				
Male	5	2.74 (1.62–3.86)	0.0%	0.855
Female	7	1.62 (1.29–1.94)	25.1%	0.22

The correlation between urine Cd concentration and the risk of osteoporosis and osteopenia was analyzed using subgroup analysis. A subgroup analysis of sex revealed that urine Cd concentration was associated with an increased risk of osteoporosis and osteopenia for both males and females (male OR = 2.74, 95% CI: 1.62–3.86, *I*^2^ = 0.00%, *P* = 0.855; female OR = 1.62, 95% CI: 1.29–1.94, *I*^2^ = 25.1%, *P* = 0.22). For the analysis of the degree of osteoporosis, urine Cd concentration was associated with an increased risk of osteoporosis (−2.5 ≤ T-score ≤ −1.0, OR = 2.03, 95% CI: 1.38–2.69, *I*^2^ = 0.0%, *P* = 0.657) and osteopenia (T-score < −2.5, OR = 1.86, 95% CI: 1.36–2.36, *I*^2^ = 47.7%, *P* = 0.033).

## Discussion

The results of our meta-analysis showed that blood Cd concentration was not associated with the risk of osteoporosis and osteopenia. However, urine Cd concentration was associated with an increased risk of osteoporosis and osteopenia.

In the 1840s, a “Itai-itai” characterized by multiple fractures and bone pain caused by Cd pollution was discovered in Japan. The patient's radiograph showed signs of false fractures of osteomalacia and severe decalcification during osteoporosis, as well as signs of proteinuria and other renal damage (35). After Cd enters the body, the kidneys and bones are the main target organs. About 50–80% of Cd accumulate in the bones and kidneys, leading to osteoporosis and also causing severe glomerular and tubular dysfunction (36). The effect on bones is considered to be the late manifestation of Cd toxicity. Regarding the mechanism of Cd specifically causing osteoporosis, Liu W et al. found that Cd can increase osteoblast apoptosis through autophagy (37). Arbon's study and other studies have also shown that Cd can directly inhibit osteoblasts and cause osteoporosis (38). Ma et al. and other studies have shown that Cd exposure significantly inhibits the differentiation of bone marrow mesenchymal stem cells, osteoblasts, and osteoclasts and promotes the occurrence of osteoporosis by promoting osteoblast apoptosis (39). In general, the pathophysiology of Cd-induced osteoporosis involves the inhibition of the accumulation of peak bone mass during growth. This adversely affects the maintenance of bone mass during bone maturation and enhances age-related osteopenia.

In an investigation of Cd-induced osteoporosis and osteopenia, Chen et al. found that high concentrations of cumulative Cd intake were associated with an increased incidence of osteoporosis and fractures in women. In men, similar trends were observed, but no statistical significance was found (40). In a study involving Japanese women, Horiguchi et al. concluded that the environmental level of Cd exposure was not enough to induce renal tubular dysfunction and would not affect bone mineral density (41). In addition to studies on adults, Sughis et al. found a consistent association between urine Cd concentration and children's bone resorption and bone demineralization in a study of children aged 8–12 years (42). In a Swedish study, Wallin et al. evaluated the effect of Cd concentration in 109 living kidneys on osteoporosis and concluded a negative correlation between kidney Cd and bone mineral density (43); however, there are very few studies on this measurement method. The current measurement method of Cd in the human body still uses blood and urine Cd concentrations as the most common biomarkers of Cd exposure. Urinary Cd mainly reflects Cd accumulation in the kidney and is also a manifestation of renal damage and osteoporosis in the later stage, whereas blood Cd shows acute and chronic exposure. The concentration of the two is essential for bone density.

There is no clear conclusion on the relationship between Cd concentration and osteoporosis and osteopenia, and there are few meta-analyses on Cd and bone health. Cheng et al. conducted a meta-analysis of Cd exposure and fracture risk and performed a subgroup analysis of urinary and blood Cd, and the results showed that Cd exposure may be a risk factor for any increased risk of fracture (44). In a meta-analysis on heavy metal concentration and osteoporosis, Jalili et al. mentioned that blood Cd is a risk factor for osteoporosis, whereas urinary Cd is not associated with osteoporosis (45). After careful comparison of reference data, there is a misclassification of urine and blood Cd data in the article, with very few studies evaluating urinary Cd concentrations; thus the article's heterogeneity makes the results questionable.

### Advantages and Limitations

To the best of our knowledge, this is the first meta-analysis to explore the relationship between blood and urine Cd concentration and osteoporosis and osteopenia. In our study, a total of 29,488 people were included, and the sample heterogeneity was small. At the same time, a subgroup analysis was conducted based on men and women and the degree of osteoporosis.

However, our research also has certain limitations. First, available research on Cd and osteoporosis is limited, which can imply that the relationship between Cd concentrations and osteoporosis and osteopenia is not sufficiently convincing. Second, observational studies have inherent limitations, such as selection bias and recall or memory bias. In addition, blood and urine Cd concentrations and the risk of osteoporosis will be affected by factors such as age. Finally, the studies included in this study may be affected by population, influence of statistical characteristics, limitations of the detection method, and other factors. For these reasons, we recommend our conclusions should be interpreted conservatively.

### Conclusion

Our research indicates that while urine cadmium (Cd) concentration may be related to increased risk of osteoporosis and osteopenia, blood Cd concentration may not. Therefore, compared to blood Cd concentration, urine Cd concentration may be more reliable as a risk factor for osteoporosis and osteopenia. This result should be interpreted with caution. Currently. research on the relationship between Cd concentration and osteoporosis and osteopenia is limited, thus, further large, high-quality prospective studies are required to elucidate the relationship between Cd concentration and osteoporosis and osteopenia.

## Data Availability Statement

The datasets presented in this study can be found in online repositories. The names of the repository/repositories and accession number(s) can be found in the article/supplementary material.

## Author Contributions

BY and HL designed the meta-analysis. HL, MZ, and JM performed the literature retrieval and the data extraction. DL and LH contributed to writing the article. All authors read and approved the final manuscript.

## Conflict of Interest

The authors declare that the research was conducted in the absence of any commercial or financial relationships that could be construed as a potential conflict of interest.
